# Biodegradable, Stretchable and Transparent Plastic Films from Modified Waterborne Polyurethane Dispersions

**DOI:** 10.3390/polym14061199

**Published:** 2022-03-16

**Authors:** Uttam C. Paul, Gözde Bayer, Silvia Grasselli, Annalisa Malchiodi, Ilker S. Bayer

**Affiliations:** 1 Smart Materials, Istituto Italiano di Tecnologia, Via Morego 30, 16163 Genova, Italy; 2 DS Bio ve Nanoteknoloji A. Ş., Lavida City Plaza 45/7, Ankara 06530, Turkey; gozde.bayer@dstrace.com; 3 GEA Mechanical Equipment Italia S.p.A., Via da Erba Edoari Mario, 29, 43123 Parma, Italy; silvia.grasselli@gea.com (S.G.); annalisa.malchiodi@gea.com (A.M.)

**Keywords:** waterborne polyurethane, polyvinylpyrrolidone (PVP), biodegradable plastic, transparent polymer, polymer hybrids

## Abstract

Waterborne polyurethane dispersions can be designed to generate highly functional and environmentally friendly polymer systems. The use of water as the main dispersion medium is very advantageous for the environment and the introduction of linear and aliphatic polyols such as polyether and polyesters in the formulations can make them highly biocompatible and susceptible to biodegradation. In this study, we fabricated biodegradable, flexible and transparent plastic films by hybridizing a waterborne aliphatic polyester polyurethane (PU) suspension with polyvinylpyrrolidone (PVP) using mechanical homogenization in water. Films were cast containing different concentrations of PVP. The hybrids containing 50 wt.% PVP (PU/PVP_50/50) were hydrophobic, stretchable, highly transparent and ductile beyond 100% strain compared to highly brittle PVP. The mechanical properties of the PU/PVP_50/50 film remained stable after repeated immersion wet–dry cycles, each lasting 2 days, and the dried films recovered their mechanical properties after each cycle. Based on a 28-day biochemical oxygen demand (BOD) test, the hybrid PU/PVP_50/50 film underwent extensive biodegradation. This simple but effective process can be very suitable in producing biodegradable ductile films with very good transparency that can serve a number of applications such as agricultural mulches, food and pharmaceutical packaging and biomedical field.

## 1. Introduction

Industrial scale emission, recycling and elimination of organic solvents are severe issues in technologies that use solvent-borne polymer dispersions [1,2,3,4]. In the last decade or so, a significant number of commercially available polyurethane (PU) adhesives and coatings have been formulated with organic solvents which are harmful to human health as well as the environment if not eliminated or recycled properly [5,6,7,8]. Today, however, many formulators and industries are shifting towards waterborne PU dispersions and in doing so, high levels of volatile organic compounds (VOCs) that might find their way into the environment are being reduced or eliminated. Waterborne PU dispersions have several advantages such as non-toxic, non-flammable and VOC compliant dispersion ingredients that can be implemented in ecofriendly adhesive and coating technologies [9,10,11]. Polymer networks in waterborne PU dispersions can be designed to exhibit a balanced combination of physical properties such as high tensile and tear strength, high elasticity, tunable hardness and abrasion resistance, good resistance to chemicals and thermal stability [12,13,14,15]. However, most waterborne PU dispersions still contain polyols derived from petroleum. In order to reduce our reliance on such a non-renewable and geo-restricted resource, bio-based polyols derived from plant origin oils are becoming more and more commonplace in PU dispersion formulations [16,17,18,19].

A major portion of waterborne PU dispersions contain linear thermoplastic polymers and have a comparatively low average molecular weight. Hence, some properties of waterborne PU dispersions may be compromised, such as water resistance, solvent resistance and elastic modulus, compared to solvent-borne PUs [20]. These could be circumvented by inducing crosslinking that can involve one-component and two-component crosslinking. In the case of a two-component crosslinking system, a crosslinker is added to the dispersion just before the application to react with the active groups in PU molecular chains at room temperature. Alternatively, in the one-component system, the crosslinker is activated during the drying process of the dispersion, after application. Waterborne PU dispersions can be used to cast polymer films with ease, however, certain technical issues such as poor miscibility or poor homogeneous distribution between the polyol and polyisocyanate can lead to poor aesthetics and appearance and even a loss of transparency [21,22]. In fact, a very recent review by Madbouly [23] extensively covered and discussed the important variables that control the thermomechanical properties and biodegradation aspects, as well as antimicrobial and biocompatibility performances of waterborne PU dispersions and their cast films that are developed for certain industrial and biomedical applications. Most waterborne PU dispersions cast films are prone to biodegradation but the rate of biodegradation can vary significantly depending on the polyol chemistry, crosslinking, hydrophilicity, use of bio-based polyols and the environment under which the biodegradation is monitored [24,25,26,27]. It is also possible to covalently bond renewable biopolymers such as polysaccharides to the polyols to enhance the biodegradation of waterborne PUs without compromising their physical and mechanical properties [28]. This is sometimes referred to as chemical hybridization, as the polyols and the natural polymer form covalent bonding [29,30].

The formulation space of waterborne PUs is very broad and this allows the formulators to design highly application-oriented dispersions. In this respect, even though increasing hydrophilic groups in the formulations has a number of advantages such as better adhesion and lamination, paintability and better biodegradation potential, water uptake levels are higher in such films and this affects the mechanical properties under humid or water-saturated conditions accompanied by some losses in transparency [31]. On the other hand, this type of PU dispersion is very attractive for novel hydrogel applications [32]. Waterborne PUs have also been used to enhance the intrinsic drawbacks of starch materials, such as poor mechanical properties and water sensitivity [33]. In this study, we modified a waterborne aliphatic polyester PU aqueous dispersion with polyvinylpyrrolidone (PVP), also commonly called polyvidone or povidone, by homogenizing the aqueous PVP solution with the PU dispersion and fabricated plastic films by casting from solutions containing different amounts of PVP. PVP is a synthetic, water-soluble, biodegradable polymer, with excellent transparency and biocompatibility but with very poor mechanical properties. Hence, modifying a petroleum origin polyester polyol waterborne PU system with a readily biodegradable and medical grade polymer without losing transparency while improving hydrophobicity and accelerating biodegradation can have significant implications in several applications, ranging from biomedical films, to general flexible packaging. We also studied the cyclic wet–dry (swollen-dry) mechanical behavior of the films and observed no degradation or changes in mechanical properties at the end of each cycle, as well as excellent repeatability and recovery, indicating that PVP does not release into the immersion media and remains chemically bound to the PU network of the films.

## 2. Materials and Methods

Polyvinylpyrrolidone (PVP, Mw 1,300,000) was purchased from Sigma-Aldrich, Milan, Italy. A commercial waterborne PU dispersion, Esacote PU-39, which is an aliphatic polyester-based polyurethane, was obtained from Lamberti S.P.A., Gallarate, Italy. All the chemicals were analytical grade and used as received without any further purification. Deionized water was obtained from Milli-Q Advantage A10 ultrapure water purification system.

### 2.1. Preparation of the PU/PVP Hybrid Films

PVP flakes were dissolved in a separate container in water at room temperature to obtain the respective solutions with a concentration of 10% (*w/v*), with the help of a vortex mixer (Heidolph D-91126, Schwabach, Germany) at 500 rpm. The homogeneous solutions were obtained after 24 h. The waterborne PU contained 36% (*w/v*) polymer in water. The commercial dispersion was diluted to 10% (*w/v*) to match the PVP solution. The solution blending of the two polymers was achieved by using a high pressure homogenizer (PandaPLUS 1000-Laboratory Homogenizer, GEA, Parma, Italy). The conditions were chosen so that the homogenization does not break down the PVP molecular weight. A minimum volume of 40–50 mL for each blend was processed at 50 bar (10,000 psi) for five cycles to produce the final dispersions and to ensure homogeneous mixing. Various desired volume ratios of the PU and PVP solutions were mixed to achieve dry films containing 0–50 wt.% of PVP in PU. The blended solutions were then sealed and allowed to rest for 10 min without stirring to remove any air bubbles. Finally, the solutions were cast in square polystyrene Petri dishes (120 × 120 mm^2^) and dried for 72 h under an aspirated hood. The films were removed from plastic Petri dishes and then cured at 130 °C for 6 h and stored under ambient conditions for further characterization. Thus, a series of hybrid films with an average thickness of 250 µm were prepared. The dry-basis PU and PVP contents for the studied hybrid films and the sample film notations are listed in Table 1.

### 2.2. Optical and Chemical Spectroscopy

UV–visible spectroscopy was performed to investigate the transparency of all samples. Rectangular-shaped specimens (2 × 6 cm^2^) were cut from the free-standing films and placed in a Varian CARY 300 Scan UV–visible spectrophotometer sample holder. The UV transmittance measurements were carried out in the wavelength range of 200–800 nm. The chemical composition and potential interactions within the blend films were characterized by an attenuated total reflectance (ATR) accessory (MIRacle ATR, PIKE Technologies, Madison, WI, USA) coupled to a Fourier transform infrared (FT-IR) spectrometer (Equinox 70 FT-IR, Bruker, Milan, Italy). The measurements were conducted within the 4000–600 cm^−1^ range with a resolution of 4 cm^−1^ and 64 repetitive scans averaged for each spectrum. Furthermore, 1H-nuclear magnetic resonance (NMR) spectra were acquired with a Varian Mercury (300 MHz) spectrometer under ambient conditions. The molar masses, M_n_, of polyols were estimated from the 1H-NMR spectra by assuming that each polyester polymer chain has two OH-terminal groups.

### 2.3. Scanning Electron Microscopy (SEM) and Atomic Force Microscopy (AFM)

The surface and cross-section morphology of the films was investigated by SEM, using a variable pressure JEOL JSM-6490LA microscope equipped with a tungsten thermionic electron source working in a high vacuum mode, with an acceleration voltage of 15 kV. Before the analysis, the samples were freeze-fractured using liquid nitrogen and placed onto the aluminum pin stub. A thin layer of gold (10 nm) was deposited on their surface using a SC7620 sputtering device (Quorum Technologies, East Sussex, UK) to avoid the surface charging effects. The EDX spectra that detects the elemental species in the samples were collected from the cross sectional portions of films and plotted as elemental spectra by the built-in software of the SEM (SmileView, Tokyo, Japan). The AFM measurement was performed on an SPA 300 HV with a SPI 3800 N controller (Seiko Instruments, Inc., Tokyo, Japan) in tapping mode. A silicon micro cantilever (spring constant 2 N m^−1^ with 300 kHz resonance frequency, Olympus Co., Tokyo, Japan) with an etched conical tip was used for the scan.

### 2.4. Thermal Characteristics Measurements

The Thermogravimetric Analysis (TGA) was carried out on the TGA Q500 device (TA Instruments, New Castle, DE, USA) to determine the thermal stability of the PU/PVP blends. A small amount (5–15 mg) of each sample was placed into platinum pans and subjected to a temperature range from 30 to 800 °C, at a heating rate of 10 °C/min. The chamber was nitrogen (N2)-purged with a flow rate of 50 mL/min. The mass loss of the sample was recorded as a function of temperature. Differential Scanning Calorimetry (DSC) was used to measure the thermal phase transitions with a Diamond-DSC (PerkinElmer, Wattham, MA, USA) instrument. Before the measurements, the instrument was calibrated using an Indium standard. The samples (5–10 mg) were sealed into the aluminum pans and subjected to a defined temperature program from −90 to 200 °C, with a heating/cooling rate of 10 °C/min under the nitrogen purge of 20 mL/min. The isothermal steps in 1 min were used to equilibrate the samples at the interval boundary temperatures. The thermal history of the samples was quenched during the first heating cycle, while data from the second heating scan was used to determine the glass transition temperature (T_g_) of the samples.

### 2.5. Measurement of Film Mechanical Properties

The mechanical properties of the PU, PVP and the PU/PVP hybrids were examined with Instron 3365 (Instron, Waltham, MA, USA) equipped with a 5 kN load cell at a cross-head speed of 5 mm/min. The films were cut into tensile bars (length of 35 mm, width of 4 mm) according to the ASTM D882–12 test standards. Elastic moduli and elongation at break values were automatically extracted by the built-in software of the instrument. The results were reported as the average values and standard deviations from five different samples. The testing was performed under ambient conditions. To observe the dry/wet elongation and robustness of PU/PVP 50/50 films, samples were soaked in water for 2 days (48 h), dried and then soaked again in water for another 2 days. This cycle was performed up to five times to investigate the wetting/drying effects on mechanical properties as well as loss of any PVP to the water. Immersion of the samples in water for 2 days was chosen based on the water absorption tests in which we measured that within 2 days, the samples reached water uptake saturation levels. The mechanical tests following wet–dry cycles were performed following the same protocol described above.

### 2.6. Wetting, Water Absorption and Swelling Properties

Static and dynamic water contact angle measurements were carried out using a Theta Optical Tensiometer (Dataphysics OCAH200) with 3 µL deionized water droplets. Static contact angles were measured with ten repetitions for each sample on different sample surface regions, and the average values were reported. To observe whether any droplets sink into the surface by bulk absorption, dynamic contact angle measurements were also conducted for up to 140 s. Water absorption tests for pure PU, PVP, and the hybrid films were carried out by soaking the samples in water. At least three specimens of 10 mg from each hybrid film were weighed and immersed in 30 mL deionized water under ambient conditions (RH 40%, 19°C). At certain time intervals, samples were removed from the water, wiped gently with tissue paper to remove the excess surface water, and reweighed. This process was performed and repeated for up to fourteen days. After the fourteenth day, no significant water absorption changes were detected, which indicated the water uptake reached its saturation and equilibrium. The water absorption percentage was then calculated based on the following formula.

(1) $$\mathrm{Water}\;\mathrm{absorption}\left(\%\right)=\frac{\left(W_{t}-W_{0}\right)}{W_{0}}\times 100$$

where W_t_ is the wet weight of the samples, and W_0_ is the original weight of the samples at RT. The swelling of the samples was measured by immersing them in water. Circular films, 24 mm in diameter, were soaked in 100 mL water. At certain time intervals, samples were removed from the water, wiped with tissue paper to remove the excess surface water, and the change in diameter was measured. This process was repeated until the swelling of the sample diameter reached its plateau, which also took fourteen days. The swelling of films was then calculated based on the following formula.

(2) $$\mathrm{Swelling}\;\left(\%\right)=\frac{\left(S_{t}-S_{0}\right)}{S_{0}}\times 100$$

where, S_t_ is the swelling diameter of the samples, and S_0_ is the original diameter of the samples at RT.

### 2.7. Biodegradation Measurements: Biological Oxygen Demand (BOD) Tests

Biodegradation measurements of the hybrid films were made following the ISO 17556:2019 standard using the OxiTop Control S6 apparatus (WTW-Xylem, Rye Brook, NY, USA) that utilizes a respirometric method to measure the oxygen demand required for the aerobic biodegradation of polymeric materials in the soil. The oxygen consumed during the degradation process was reported as the biochemical oxygen demand (BOD) in milligrams of captured oxygen per mass unit of the tested hybrid films [34]. The OxiTop Control S6 apparatus comprised six glass bottles with 510 mL volume equipped with rubber quivers and measuring heads, which recorded the BOD values. The measuring heads also recorded the pressure within a range of 500 to 1350 kPa, with an accuracy of 1% that can operate between 5 to 50 °C.

The measurements were controlled by WTW™ OxiTop™ Controller (OxiTop OC 110) that can monitor up to 100 measurements simultaneously. The controller was connected to a computer via a WTW Achat OC PC communication program with an interface cable AK 540/B for further data processing and documentation of measurement data. An organic garden soil (Nif Organik Bahçe Toprağı) rich in humus with a moisture content of about 5% and pH 6 with a 1–3 mm grain size was purchased from Nif Organik, Izmir, Turkey and used as the biodegradation medium. Pure PU and hybrid films weighing 200 mg were mixed into 200 g of soil, 100 g of distilled water was added later, the system was gently mixed and hermetically sealed in the glass bottles and incubated at 20 ± 0.2 °C for 28 days. The biochemical oxygen demand (BOD) for each glass bottle was determined from Equation (3) below, in which a control experiment that contains only the soil sample is required.

(3) $${\mathrm{BOD}}_{S}=\frac{{\mathrm{BOD}}_{X}-{\mathrm{BOD}}_{G}}{C}$$

where S is the number of measurement days, BOD_S_ stands for biochemical oxygen demand of the sample film at end of S days (mg/L), BOD_X_ designates the biochemical oxygen demand of the measuring system (bottle with sample and soil) (mg/L), BOD_G_ means the biochemical oxygen demand of soil without a sample (mg/L) and C is the sample concentration in the tested system (mg/L). The degree of biodegradation of the polymeric material can be determined from Equation (4) as:

(4) $$D_{t}=\frac{{\mathrm{BOD}}_{S}}{\mathrm{TOD}}\times 100\%\;$$

where D_t_ is the degree of oligomeric polyhydric alcohol biodegradation (%), TOD is the theoretical oxygen demand (mg/L) that can be calculated from Equation (5):

(5) $$\mathrm{TOD}=\frac{16\left[2C+0.5H-O\right]}{M_{n}}\;$$

where C, H, O are the mass shares of elements in the polymer system and, M_n_ is the molecular weight of it (g/mol).

## 3. Results

### 3.1. Film Morphology and Transparency

**Figure 1:**
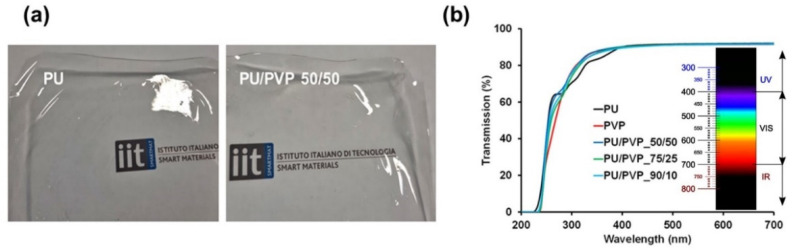
(**a**) Photographs of a transparent films comprising 100% PU (left) and PU/PVP_50/50 (right), (**b**) UV-Vis spectra of the blend films including pure PU and PVP polymers. The inset shows the UV-Vis spectrum extending into the IR region.

The waterborne aliphatic polyester PU forms a transparent film that can be peeled off easily from polystyrene Petri dishes (Figure 1a, left panel). Similarly, the waterborne PU could be mixed with the PVP solution at any ratio, forming transparent hybrid films, see for instance Figure 1a (right panel). Particularly, the PU/PVP hybrids were very interesting and completely transparent as shown in Figure 1b. The transmission exceeds 90% at around 350 nm, indicating that the films are transparent starting from the UVA and UVV wavelengths (320–400 nm) to the visible red range (~740 nm). All the films block transmission of far UVC light (207 to 222 nm) that is considered to be an important wavelength range that efficiently kills pathogens potentially without harm to exposed human tissues [35]. In fact, between 300 to 400 nm, the hybrid films display slightly better transparency compared to the pure PU film, as shown in Figure 1b.

**Figure 2:**
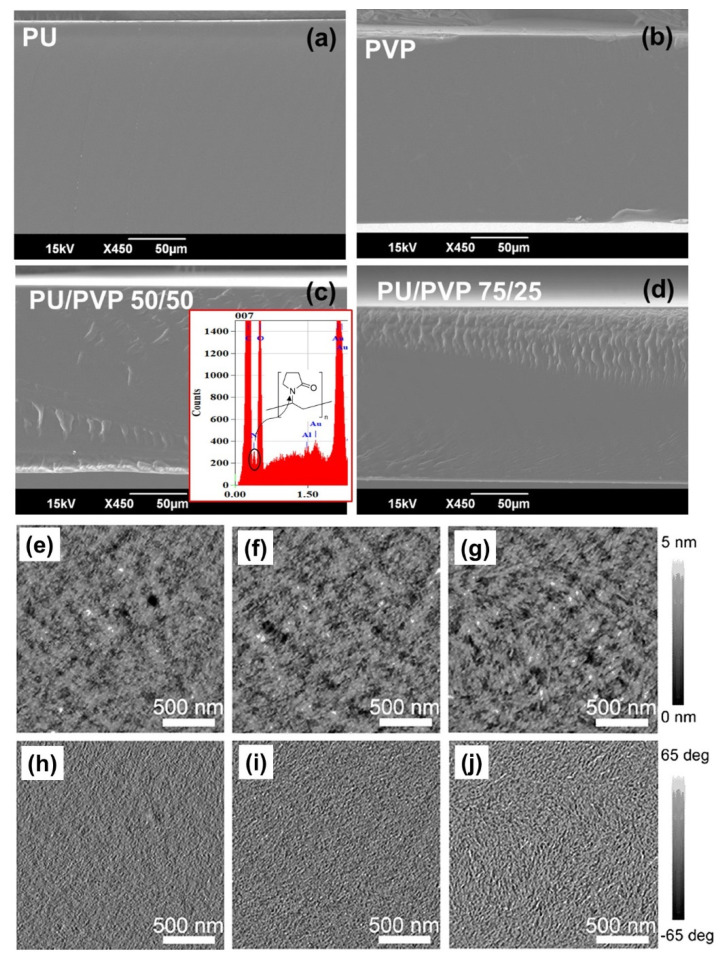
SEM cross section images of (**a**) pure PU film, (**b**) pure PVP film, (**c**) PU/PVP_50/50 hybrid film and (**d**) PU/PVP_75/25 hybrid. AFM height and phase images for PU/PVP_90/10 (**e**,**h**), PU/PVP_75/25 (**f**,**i**) and PU/PVP_50/50 (**g**,**j**).

The homogeneity of the hybrid films is also evident if one inspects the cross sectional SEM film images in Figure 2. Both the pristine and the hybrid films are featureless. The EDX image of the PU/PVP_50 film shown in Figure 2c as the inset shows a noticeable nitrogen element signal due to povidone polymer that was not as intense and distinguishable in the PU polymer film spectrum (not shown for brevity).

Compared to other biodegradable transparent hybrid films [36], the present hybrid films contain much higher amounts of the most biodegradable component (i.e., 50 wt.% PVP) without compromising film homogeneity and transparency. The surface SEM images of the hybrid films were featureless but their morphology was inspected using AFM, instead, as shown in Figure 2e–j. The morphology (height) and phase images are typical of amorphous polymer blends, indicating good nanoscale miscibility between the polymers.

### 3.2. Chemical Properties of the Films

**Figure 3:**
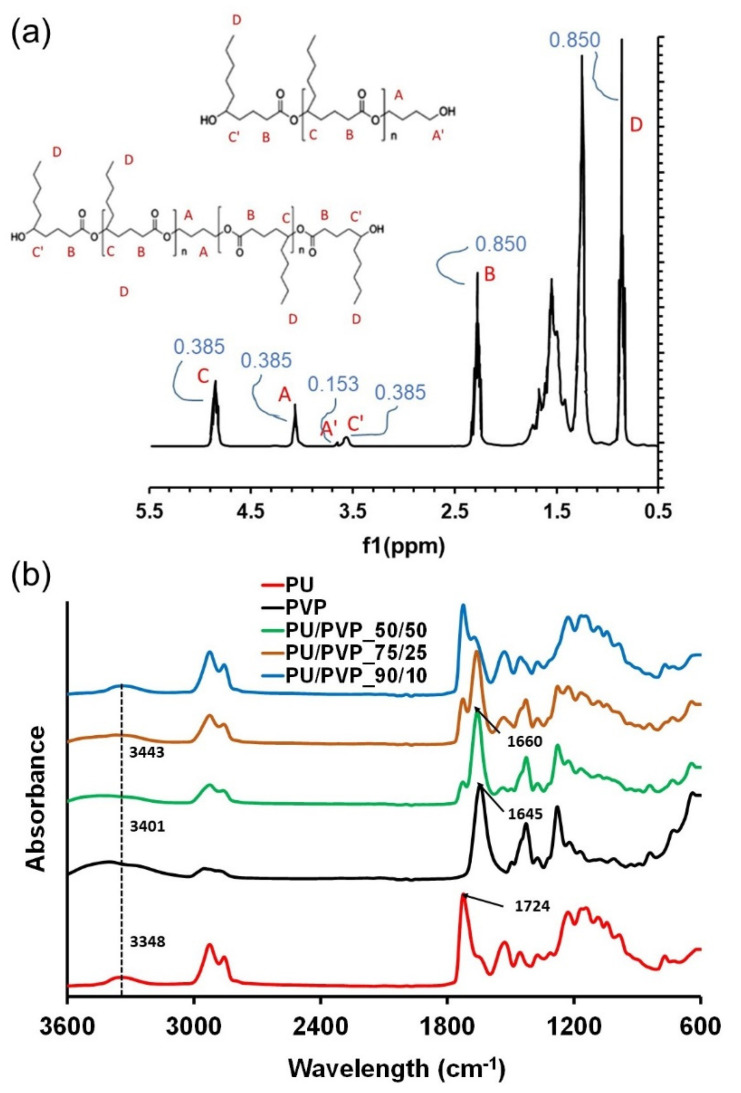
(**a**) 1H-NMR spectrum of the PU polymer structure indicating an aliphatic polyester polyol system based on the ring opening polymerization of 7-Butyl-2-oxepanone type polyol (**b**) FTIR spectra of the pure polymers and all the hybrid films shown in Table 1.

The waterborne PU dispersion used in this study is based on an amorphous polyester diol. There is general agreement that the biodegradability of PUs can be realized by employing aliphatic polyesters as the soft segments [37, 38, 39]. However, if these aliphatic polyesters are based on semi-crystalline polymers such as polycaprolactones, polyadipates, polysuccinates, the PUs can have limited elasticity and transparency. The 1H-NMR spectrum of the PU suspension is shown Figure 3a. The spectrum indicates aliphatic polyester with a dihydroxyl-terminated structure. Not all of these hydroxyl groups reacted with the initiator, since the signal H_a_ (CH_2_–OH) is visible in Figure 3a. This can be eliminated if the PU film is aged or thermally conditioned for a longer time. From the results of the signal ^1^H-NMR, we estimate that the polyester part is based on the ring opening polymerization of 7-Butyl-2-oxepanone type polyol with an estimated molecular weight of about (M_n_ = 3000 g mol^−1^). The estimated aliphatic polyester chemical structure is shown as inset in Figure 3a.

In Figure 3a, the peak integration results are also displayed. The peak integration of 1H NMR signals may not be very accurate due to peak splitting, which is affected by neighboring C-H bonding, however, the area of a given peak in a 1H NMR spectrum is proportional to the number of (equivalent) protons giving rise to the peak. In Figure 3a, the integration values extracted from the software are less than 1.0, were normalized by the lowest number and rounded to the next integer to calculate the number of H atoms that are marked in the plausible polyol formula of the PU.

FTIR-ATR analysis was used to identify potential changes in the functional groups upon the hybridization of PU and PVP polymers. The C=O and N–H groups present in PU can form a hydrogen bonding interaction with polar groups of PVP. As shown in Figure 3b, the bare PU was characterized by a peak at around 3348 cm^−1^ due to the existence of the N–H stretching vibration. The carbonyl absorption peak located at 1724 cm^−1^ can be attributed to a free carbonyl stretching vibration. In the case of PVP, the hydroxyl peak can be seen at 3401 cm^−1^. Upon hybridization with PVP, a new peak appeared at 1650 cm^−1^ in all the hybrids, which corresponds to the typical carbonyl group in the pyrrolidone ring of PVP. The hydroxyl (O-H) group shifting from 3401 cm^−1^ of PVP to 3443 cm^−1^ for PU/PVP 50/50, indicates that the hydrogen bonding interactions in the blends are strong, as shown in Figure 3b. The FTIR data do not indicate the formation of new polymers but suggest the presence of some covalent and hydrogen bond interactions such as the peaks near 1650–1660 cm^−1^ related to amide-lactam interactions of RCONNR_2_ type and signal shifts in the region 3300–3450 cm^−1^.

### 3.3. Thermal Properties of the PU/PVP Hybrids

**Figure 4:**
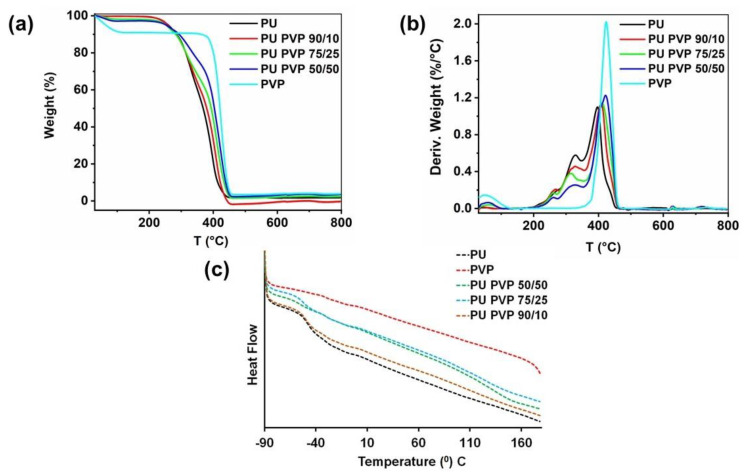
**(a**) TGA, (**b**) DTGA, and (**c**) DSC plots of pure and the hybrid films shown in Table 1.

The thermal degradation of the pristine polymers and the hybrids was studied by thermal gravimetric analysis (TGA), as shown in Figure 4. The hygroscopic character of PVP polymer is evident in Figure 4a, in which about 8% adsorbed water loss was measured until the system temperature reached 100 °C. Pure PU displayed no hygroscopic features and demonstrated the onset of degradation temperature at 395 °C, while PVP demonstrated this at 425 °C. As Figure 4b indicates, the PU polymer has a minor weight loss temperature of about 325 °C. It is generally known that the derivative TGA curves of polyurethanes show several different degradation stages between 220–370 °C. The range 230–340 °C is associated with the degradation of the hard phase (crosslinks); and the range 350–370 °C indicates dissociation of the soft segments. The onset of degradation temperatures of PU/PVP hybrids was observed at 422, 410, and 408 °C for 50/50, 75/25, and 90/10 PU/PVP hybrids, respectively. Thus, it can be stated that PVP improved the thermal degradation resistance of the PU polymer. Figure 4c shows the differential scanning calorimetry (DSC) thermograms of PU, PVP, and the hybrids. The curves indicate that the glass transition temperature (T_g_) value for the pristine PU was found to be −47.3 °C, while PU/PVP hybrids exhibited T_g_ values of −49.2 °C, −52.8 °C, and −57.1 °C for PU/PVP_90/10, 75/25, and 50/50 samples, respectively. This indicates that the T_g_ of PU/PVP blends gradually decreased with the increase in PVP content, indicating more molecular mobility within the hybrid films.

### 3.4. Mechanical Properties of the Films

**Figure 5:**
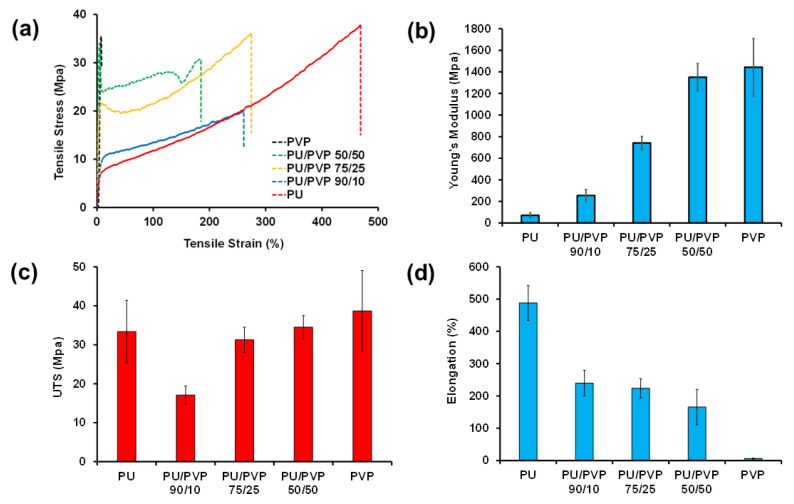
Mechanical properties of PU, PVP, and the hybrids. (**a**) Typical stress–strain curves, (**b**) Calculated elastic or Young’s moduli (MPa), (**c**) Ultimate tensile strength (UTS) (MPa), and (**d**) Elongation at break (%).

Figure 5 shows the stress–strain measurement results for all the films studied. Specifically, Figure 5a shows the typical tensile stress–strain curves where a sharp contrast between the PVP and the PU film mechanical responses can be seen. Elastic (Young’s) moduli of pure PU and PVP polymers extracted from the curves are 33.4 ± 8.0 MPa, 1.4 ± 0.25 GPa, respectively. This makes PVP an extremely rigid polymer compared to the much softer PU. In fact, as seen in Figure 5a, the pure PU can stretch up to 500% before breaking. Figure 5b shows the elastic moduli of all the hybrid films in which the elastic modulus of the hybrid PU/PVP_50/50 matches that of pure PVP while its elongation at the breaking value exceeds 150%. The ultimate tensile strength (UTS, maximum stress before break) values of both pure PU, PVP are similar, as shown in Figure 5c. The hybrid PU/PVP_90/10 has the lowest UTS value (Figure 5c). According to Pukinszky and Tudos [40] Young’s modulus of polymer blends is less sensitive to interaction and morphological changes than the yield and ultimate properties. They showed that UTS is very sensitive to polymer chain morphologies in blends as well as interfacial tensions between the polymer chains. These two parameters can change easily as a function of the respective polymer concentrations, as may be the case for PU/PVP_90/10 [40]. Inspecting Figure 5c further shows that the hybrids PU/PVP_75/25 and PU/PVP_50/50 feature also yield stresses and stress–strain behaviors that are very indicative of “hard and tough” thermoplastic polymer response [41]. Regarding the elongation at break values reported in Figure 5d, pure PU shows a quite high value of 488.1± 53.8%, whereas, for the PVP, the elongation is only 6.1± 1.5%. However, even the hybrid film with 50 wt.% PVP content still shows about 200% elongation before break. In summary, we can conclude that the hybrid system formed by PU/PVP_50/50 is a unique example of a tough but stretchable film based on a highly rigid biopolymer PVP.

### 3.5. Wetting and Long-Term Water Uptake Properties

**Figure 6:**
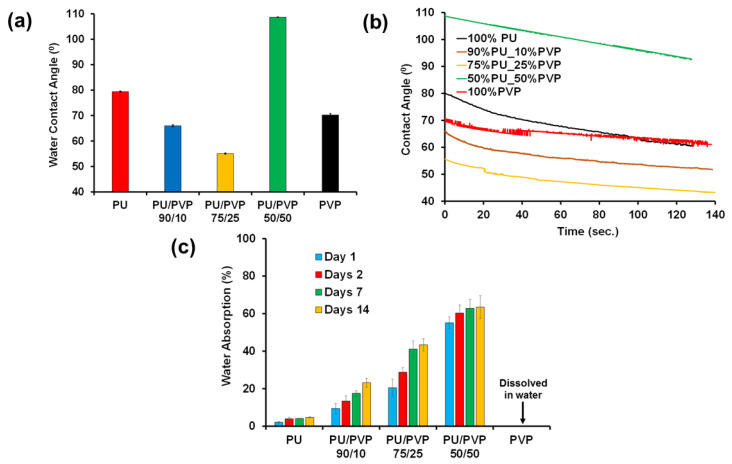
(**a**) Static water contact angle, (**b**) dynamic water contact angle, and (**c**) percent water absorption data of pure polymers and the hybrid films.

The surface hydrophilicity of the films, including the pure polymers, was analyzed by static and dynamic water contact angle measurements as shown in Figure 6a. The pure PU film exhibited a contact angle of 79.4 ± 0.20, indicating a relatively hydrophobic nature. On the pure PVP film, the contact angle was measured to be 70.2 ± 0.50. The hybrid films PU/PVP_90/10, and PU/PVP_75/25 exhibited lower contact angles and were more hydrophilic than the respective pure polymers, as shown in Figure 6a,b. In the case of the PU/PVP_50/50 hybrid film, the contact angle was much higher at 110°. This indirectly suggests that the polymers interact with strong hydrogen bonds, leaving no interacting polar groups on the surface of the films. Similar systems were also reported earlier, in which two hydrophilic polymers were hybridized, thereby forming hydrophobic composite films [42]. The dynamic water contact angle measurements reported in Figure 6b show that the contact angles change slightly over a period of 2 min under ambient conditions which can be attributed to the evaporation of the droplets rather than droplet absorption into the film or a sudden change in wetting state [42]. Separately, the water absorption tests for all the films that extend to a two-week period are reported in Figure 6c. The pure PU film was very stable and its water uptake remained below 5% compared to pure PVP that completely dissolves in water after a couple of hours. Increasing the PVP content in the films increases the water uptake values gradually up to about 60% for the PU/PVP_50/50 hybrid film at the end of the 14 day period. No dissolution whatsoever was observed at the end of the measurements that proves the formation of an interacting blend between the PVP and PU polymer chains.

### 3.6. Swelling and Mechanical Film Recovery after Wet/Dry Cycles

**Figure 7:**
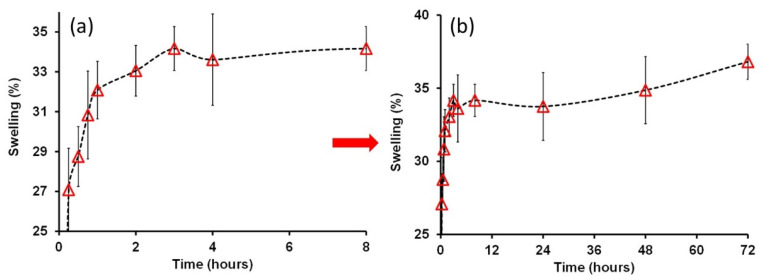
Percent swelling measurements for the PU/PVP_50/50 film monitored for up to three days. (**a**) The initial 8 hours is displayed in detail and (**b**) the full 3-day plot is presented.

We chose the PU/PVP_50/50 film in order to conduct further tests such as swelling and to detect changes in its mechanical properties as a function of wet–dry cycles when the film is immersed in water. Figure 7a shows percent swelling in the film as a function of water immersion time in hours. The maximum swelling occurred at around at 34% after 4 h in water. Monitoring further changes in percent swelling up to 3 days continuously, as shown in Figure 7b, designates that the percent swelling does indeed saturate at around 35% and does not change any further when immersed in deionized water. Moreover, as seen in Figure 7, the films do not transform into a hydrogel state, since the degree of swelling in hydrogels can be in excess of 1000% [43].

**Figure 8:**
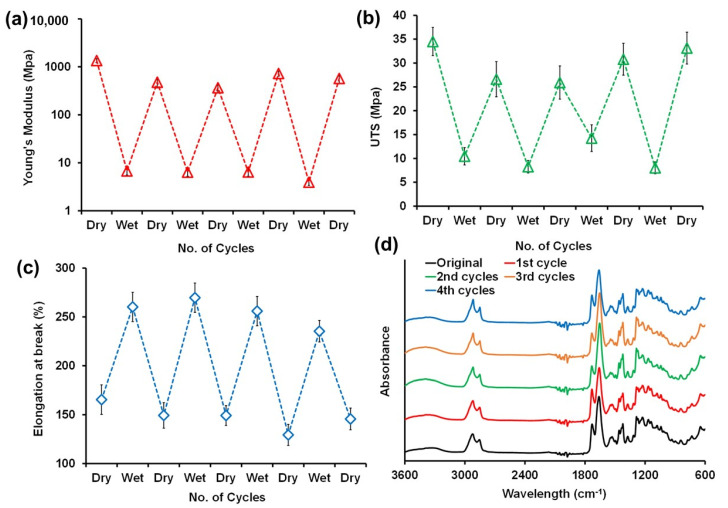
Mechanical properties of PU/PVP_50/50 film in response to wet–dry cycles. (**a**) Changes in elastic modulus, (**b**) changes in UTS, (**c**) changes in elongation at break values and (**d**) FTIR spectra after each wet–dry cycle indicating no chemical changes in the film due to cycling tests.

The same film was also subjected to several wet–dry cycles as shown in Figure 8. More specifically, we measured the changes in the elastic modulus (Figure 8a), UTS (Figure 8b) and elongation at break (Figure 8c) values of the film when it was soaked in water for two days continuously, dried and soaked again for two days in a repeated fashion. This process was continued for up to four repetitive cycles and the mechanical properties of the film samples were measured in both wet and dry states. From Figure 8a–c it can be seen when the films are soaked in water the elastic modulus declines to about 7 MPa when wet and the UTS declines by three times to about 10 MPa. Elongation at break values, on the other hand, increase by about two times when wet. These wet-state values remained practically constant at the end of each cycle. The pristine hybrid film has an elastic modulus of about 1.2 GPa as was also shown in Figure 5b. After the first wet–dry cycle the dry-state elastic modulus of the film declined to about 850 MPa and remained practically stable at this value for the rest of the wet–dry cycles. This change could be attributed to the fact that water can establish permanent or shielding intra- and inter-macromolecular hydrogen bonds and dipole–dipole interactions with biopolymers that can cause a plasticizing effect [44]. This is not necessarily achieved by water immersion and does not require a lot of water but even atmospheric moisture can cause such a permanent plasticization effect in biopolymers over time, also known as bound water [45].

Potential chemical changes in the film due to wet–dry cycles were monitored by FTIR, as shown in Figure 8d. As can be seen, no new peaks or shifts in peaks were noticed in the FTIR spectra at the end of each cycle. As such, continuous immersion cycles in water could only effect the ductility of the films due to partial plasticization. This observation is quite promising and quite impossible to attain using the pure PVP polymer or films alone due to their poor resistance to water [46].

### 3.7. Biochemical Oxygen Demand (BOD) Analysis of the Films

Plastic pollution in the environment is an ever increasing problem, including plastics in aqueous environments such as our oceans. In fact, biopolymers and bioplastics will help curb plastic pollution in oceans but biodegradable polymers including modified biodegradable polyurethanes can also be used to restore aquatic life such as reconstruction of coral reefs [47]. As mentioned earlier, aliphatic polyester polyol-based polyurethanes are susceptible to biodegradation depending on molecular weight, crosslinking and degradation conditions [48,49,50].

**Figure 9:**
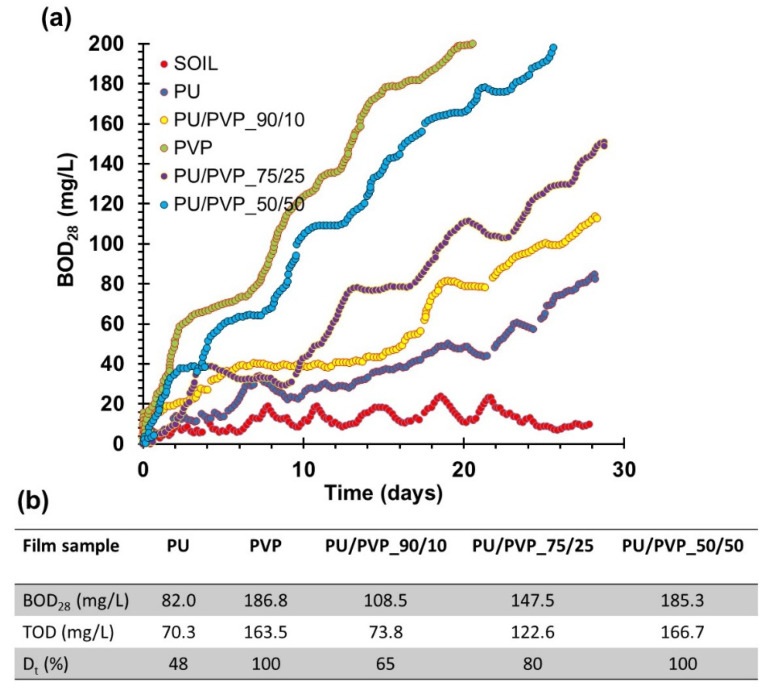
(**a**) Biochemical oxygen demand measurements for pure polymers and the hybrid films, including the control soil sample. (**b**) Estimated theoretical oxygen demand values and biodegradation percent for the films.

The biodegradation potential of the films including the pure polymers and the organic garden soil were tested using the OxiTop Control S6 apparatus to understand susceptibility of the films to home compost-like conditions. The sample films that weighed about 200 mg were monitored for 28 days. The amount of dissolved oxygen needed by aerobic biological organisms to break down the film samples is plotted in Figure 9a. The BOD value is most commonly expressed in milligrams of oxygen consumed per liter of sample during the whole incubation period in the bottles. The final BOD values were registered for each film at the end of 28 days. The mass shares of carbon, hydrogen, and oxygen for each polymer/polyol were estimated based on am elemental analysis/polymeric structure of each polymer and the results are tabulated in Figure 9b. The estimates were used to calculate the theoretical oxygen demand TOD (mg/L) and the percent degradation of each film (D_t_, %) in Figure 9b. The results are quite encouraging and even the pure PU film displays a 50% degradation rate at the end of roughly one month of testing. In fact, the measured BOD values in Figure 9b were higher than the TOD values for all films tested. This suggests that the degradation of the films can positively affect the aerobic microorganism (organism proliferation) growth in the testing system [51,52]. It is important to note that this test does not exactly indicate that the films are completely biodegradable, since it does not replicate many different environmental conditions; however, it shows the susceptibility of the films to biodegradation that is found to be quite high. Further tests that comply with home and industrial compost standards need to be conducted.

In summary, we can conclude that the proper hybridization of polyurethanes with biodegradable polymers can significantly increase the potential applications of certain biopolymers with highly disadvantageous inherent properties such as mechanical rigidity and poor resistance to water and humidity while preserving other properties such as transparency or functionality [53], including inherent antioxidant potency [54] that could be suitable for a variety of packaging applications.

## 4. Conclusions

In this study, we combined an aliphatic polyester waterborne polyurethane (PU) polymer with polyvinylpyrrolidone (PVP) in water using mechanical homogenization. Upon solution casting, biodegradable, flexible and transparent hybrid films were made. Hybrid films that contained 50 wt.% PVP (PU/PVP_50/50) were stretchable and ductile beyond 100% strain compared to highly rigid and brittle PVP polymer. The hybrid films did not display the inherent hygroscopic features of the PVP polymer and showed very stable thermal degradation profiles. The films were thermally stable and the hybrid PU/PVP_50/50 film was more hydrophobic than all the polymers and combinations studied. Upon immersion in water, swelling did not exceed 35%, which indicated the hybrid films did not transform into hydrogels that normally display swelling rates exceeding 1000%. The mechanical properties of the PU/PVP_50/50 film did not practically degrade after long-term (8 days) water immersion wet–dry cycles and the dried films recovered their mechanical properties after each cycle. No PVP leached into the water during the cyclic immersion experiments. Water partially plasticized the films, however, upon drying, the original elongation at break values were restored. Very promising biodegradation results were obtained from the hybrid films based on a 28 day biochemical oxygen demand test using an organic garden soil/water system. Hence, we can claim that the films are suitable for a number of applications such as agricultural mulch, food and pharmaceutical packaging and in the biomedical field.

## Figures and Tables

**Table 1:** Sample names for different hybrid films and their respective PVP and PU contents on dry basis.

Sample	PVP (wt.%)	PU (wt.%)
PU	0.0	100.0
PVP	100	0.0
PU/PVP_50/50	50	50
PU/PVP_75/25	25	75
PU/PVP_90/10	10	90
